# Heart rate dynamics and asymmetry during sympathetic activity stimulation and post-stimulation recovery in ski mountaineers—a pilot exploratory study

**DOI:** 10.3389/fspor.2024.1336034

**Published:** 2024-02-29

**Authors:** Jakub S. Gąsior, Maciej Gąsienica-Józkowy, Marcel Młyńczak, Maciej Rosoł, Robert Makuch, Rafał Baranowski, Bożena Werner

**Affiliations:** ^1^ Department of Pediatric Cardiology and General Pediatrics, Medical University of Warsaw, Warsaw, Poland; ^2^ Higher School of Rehabilitation in Warsaw, Warsaw, Poland; ^3^ Faculty of Mechatronics, Institute of Metrology and Biomedical Engineering, Warsaw University of Technology, Warsaw, Poland; ^4^ Department of Physical Education, Kazimierz Pulaski University of Technology and Humanities in Radom, Radom, Poland; ^5^ Department of Heart Rhythm Disorders, National Institute of Cardiology, Warsaw, Poland

**Keywords:** heart rate variability, symbolic dynamics, heart rate asymmetry, sympathetic stimulation, recovery, athletes

## Abstract

There is a lack of studies on non-linear heart rate (HR) variability in athletes. We aimed to assess the usefulness of short-term HR dynamics and asymmetry parameters to evaluate the neural modulation of cardiac activity based on non-stationary RR interval series by studying their changes during sympathetic nervous system activity stimulation (isometric handgrip test) and post-stimulation recovery in professional ski mountaineers. The correlation between the changes in the parameters and the respiratory rate (RespRate) and also the duration of the career was analyzed. Short-term (5 min) and ultra-short-term (1 min) rates of patterns with no variations (0V), number of acceleration runs of length 1 (AR1), and short-term Porta's Index were greater, whereas Guzik's Index (GI) was smaller during sympathetic stimulation compared to rest. GI increased and the number of AR1 decreased during recovery. Greater increases in GI and RMSSD were associated with greater decreases in RespRate during recovery. Greater increases in RespRate from rest to short-term sympathetic stimulation were associated with greater increases in 0V (Max-min method) and AR1 but also with greater decreases in decelerations of short-term variance and accelerations and decelerations of long-term variance. Greater increases in 0V (Max-min method) and number of AR1 during sympathetic stimulation were associated with a shorter career duration. Greater decreases in these parameters during recovery were associated with a longer career duration. Changes in measures of HR dynamics and asymmetry, calculated based on short-term non-stationary RRi time series induced by sympathetic stimulation and post-stimulation recovery, reflected sympathovagal shift and were associated with condition-related alterations in RespRate and career duration in athletes who practice ski mountaineering.

## 1 Introduction

Recognizing and modifying the cardiac autonomic activity of individual athletes could be helpful in prescribing an adequate training load, controlling the onset of fatigue, improving physical performance, and achieving better athletic results (1–4). Athletes with higher vagal modulation at rest confer greater tolerance to intense exercise regimens (5) whereas sympathetic predominance before and/or during real-world sports competition may be advantageous for performance (6).

Evaluation of cardiac autonomic nervous system modulation by analysis of heart rate (HR) and its variability (HRV) is becoming increasingly popular in sports science (1–4). During the transition from rest to exercise, HR initially accelerates due to a decrease in parasympathetic nervous system activation, followed by an increase in sympathetic tone. Recovery is characterized by an initial HR deceleration based on a sudden vagal reactivity followed by a gradual reduction in sympathetic tone (7, 8). HRV in athletes is routinely analyzed using linear methods, which are adequate to assess autonomic activity at rest based on stationary-confirmed RR time series (9–11). Indeed, spectral analysis is not appropriate to characterize the complex dynamics of HR modulation from non-stationary interbeat interval series obtained during short-term unstable conditions (such as progressive physical effort or recovery period) that lead to, inter alia, respiratory rate alterations (12–17). Popular in sports science, the vagally-mediated time domain the root mean square of successive RR differences (RMSSD) has been calculated based on shorter segments of 5 min exercise and post-exercise recovery (18, 19), but its potential lack of validity for non-static conditions (20) and as a measure of parasympathetic reactivity during slow deep breathing has recently been described (21).

Nonlinear methods that quantify the qualitative features of complex dynamics have opened new opportunities for monitoring cardiac autonomic regulation in non-stationary environments (22–26). The short-term scaling exponent alpha1 of detrended fluctuation analysis is based on the fractal correlation properties of the beat-to-beat cardiac pattern (27). Numerous studies have shown its suitability for describing cardiac autonomic regulation based on non-stationary data from different exercise times, intensities, and modalities (12, 13, 28, 29). Other nonlinear methods may have potential for use in sports medicine, but their usefulness for athletes has rarely been studied so far (14, 30, 31). The symbolic dynamics method, based on the classification of three consecutive RR intervals (RRi) and the estimation of the rate of specific pattern categories (32), represents an alternative to linear spectral analysis for the investigation of sympathovagal interactions (32, 33) from short and very short term recordings (14), and is also independent of changes in respiratory rate (34). The rate of patterns with two dissimilar variations was found to correlate with active vagal control and sympathetic withdrawal (33, 35, 36), while those with no variations correlated with the relevance of sympathetic control (33, 35). Heart rate asymmetry (HRA), defined by different approaches using the Poincaré plot, reflects the unbalanced contribution of HR accelerations and decelerations to short-, long-term, and total HRV, but has mostly been studied in subjects in stationary conditions (37–44). An asymmetric pattern with bradycardic runs (sequences characterized by prolongation of the heart period) shorter than tachycardic runs (i.e., the heart decelerates more rapidly than it accelerates) has been observed when sympathetic regulation is predominant (43, 45). The dependence of HRA on alterations in respiratory rate is under debate (46–48).

The purpose of this study was twofold: (i) to assess group and individual changes in HR dynamics and asymmetry during short-term inherently non-static conditions, i.e., during sympathetic nervous system activity stimulation (SNSa stim.) and post-stimulation recovery; and (ii) to verify whether these changes are associated with respiratory rate changes and professional career duration in athletes who practice ski mountaineering—a new Olympic, rapidly growing but still of little scientific interest (49), winter sport (50, 51).

## 2 Materials and methods

### 2.1 Population

A total of 11 elite (52) ski mountaineering athletes (6 of whom were women) participated in the study. Inclusion criteria were: being an active athlete (53) currently in possession of a license from the National Mountaineering Association; being in the pre-season period; accepting and complying with the measurement rules (Procedures and measurement conditions are below); absence of diseases and/or regular use of medications affecting the cardiopulmonary system and/or interfering with the autonomic nervous system. The study was approved by the Ethics Committee of the Higher School of Rehabilitation in Warsaw (Poland) (No. 103/2021, 20.02.2021) and followed the rules and principles of the Declaration of Helsinki. All athletes were informed about the aims of the study, the measurement protocol, and the potential risks and benefits of the study by conversation 3 months before the measurements and by e-mail with instructions and procedures 2 weeks before the experiment. All athletes provided written informed consent prior to data collection.

### 2.2 Procedures and measurement conditions

The athletes were instructed to maintain normal sleep behaviors (as usual during the 5 days before the examination), to refrain from physical activity, caffeine, and alcoholic beverages on the day before and the day of the study, to avoid smoking, to eat a normal, usual light breakfast, and to use the toilet (if necessary) on the day of the study before the examinations. The examinations were carried out at least 1 h after breakfast at home and before lunch. All examinations were performed in a quiet, bright medical room with a stable, controlled temperature and humidity. Body mass (kilograms) and height (meters) were measured to calculate the body mass index. In order to stabilize HR and respiratory rate, the participants were asked to lie in the supine position for ∼10 min before the beginning of the appropriate ECGs (used to calculate HRV). Each athlete underwent three consecutive ECG examinations: 1st (6 min) under established, controlled measurement conditions (Rest), 2nd (6 min) during sympathetic nervous system activity stimulation by static (isometric) exercise (SNSa stim.) (54, 55) and 3rd (6 min) as post-stimulation recovery. To stimulate SNSa, the subjects were asked and encouraged to continuously squeeze the Saehan hydraulic hand dynamometer (model SH5001, Saehan Corporation, Masan, South Korea, second handle position) at 30% of their maximal voluntary contraction using their dominant hand, maintaining the adequate value controlled by two researchers. The athletes were asked to refrain from speaking or moving during the ECG examinations.

### 2.3 RRi data acquisition

A twelve-lead ECG (Custo cardio 100 12-channel PC ECG system, sampling frequency—1,000 Hz; Custo med GmbH, Ottobrunn, Germany) was used to register RRi. The RRi of athletes with confirmed sinus rhythm and no cardiac abnormalities were exported from the ECG software as.xlsx to identify and correct artifacts. Technical artifacts (e.g., due to sweating, movement, and/or poor electrode fixation) were identified as one of seven types of errors according to the identification procedure (56, 57). Technical and physiological artifacts (ectopic beats, premature atrial and/or ventricular beats) present in the ECG signal were replaced by interpolated RRi from adjacent RRi (58, 59).

### 2.4 RRi stationarity assessment

Stationarity (consistent stability over the recording period) of the RRi data series—an important requirement for HRV analysis (60, 61)—was verified before HRV analysis (details in Statistical analysis and Supplementary Materials).

### 2.5 Respiratory rate monitoring

In the present study, athletes performed spontaneous breathing (62). The abdomen, thorax, and neck were video-recorded (Sony® HDRAS20 Action Camera) to calculate the respiratory rate (RespRate), which was determined from the number of respiratory cycles counted. The beginning of each respiratory cycle was defined as the end of the inspiratory phase when the diaphragm was at the apex.

### 2.6 HR and time-domain HRV analysis

The corrected RRi were imported into Kubios HRV Standard 3.4 software (University of Eastern Finland, Kuopio, Finland) (63) to calculate HR and time-domain HRV. Imported RRi series were not further corrected for artifacts using an integrated Kubios software tool. The RRi series were not detrended to avoid loss of physiological information related to signal non-stationarity (61). Mean RR interval (mRR), mean HR (HR), minimal—HR_min_ and maximal—HR_max_, standard deviation of normal-to-normal RRi (SDNN), root mean square of successive RR interval differences (RMSSD), log-transformed RMSSD (lnRMSSD) and pNN50 (percentage of RR intervals differing >50 ms from the preceding one) were calculated based on 5 min recordings for all analyzed conditions.

### 2.7 Symbolic dynamics

Screened and corrected 5-min RR series were imported for analysis into PyBiOS software (64) from ASCII text files to calculate symbolic dynamics indices. Imported RRi series were not post-processed at all (i.e., corrected for artifacts, segmented, filtered, or detrended using software tools). Symbolic dynamics indices were obtained using three transformation methods: the σ method, the Max–min method, and the Equal probability method (65, 66). In the σ method, three levels were defined using the following quantization lines: the signal average (*μ*), the signal average shifted up by a factor a, that is, (1 + *a*) *μ*, and the signal average shifted down by a factor a, that is, (1−*a*) *μ*. The parameter *a* (sigma rate) was set to 0.05 (67). In the Max–min method, the series of RR intervals was converted into a series of symbols by a uniform quantization of six levels (quantization level: 6). This means that six equal ranges were defined from the minimum to the maximum value within the series [*l *= (max(*x*i)—min(*x*i)]/ξ, quantization level *ξ *= 6, and each value in the original series was converted into a symbol (0–5) (32, 33). The Equal probability method divides the full range of the signal into quantization levels ensuring that each level contains the same number of points. Thus, if the signal has length *L*, each level will have *L*/quantization level samples. If *L* is not a multiple of the quantization level, the number of points can vary by one within the levels (66). The transformation was used with two different quantization levels: 4 (*q *= 4) and 6 (*q *= 6) allowing a direct comparison with the σ method and the Max–min method, respectively. For all three methods, all sequences of three consecutive symbols (words) are classified into one of four families: 0V (zero variation)—three symbols are the same (examples: {1,1,1} and {5,5,5}); 1V—only one variation (examples: {1,1,2} and {3,3,0}); 2LV (two like variation), representing sequences with two variations in the same direction, that is, the symbols are all different and form an increasing or decreasing ramp (examples: {0,3,5} and {2,1,0}); 2UV (two unlike variation), where the symbols vary twice, in opposite directions, forming a peak or a valley (examples: {1,2,0} and {3,0,3}). The percentages of words classified in each family were used for the analysis of the dynamics of the series. In this study, 0V and 2UV are presented due to their physiological interpretation (32, 33, 35, 65, 66, 68).

### 2.8 HR asymmetry

For HRA analysis, the free-of-charge HRAExplorer software available at https://hraexplorer.com (accessed on 1 December 2022) was used. To quantify HRA Guzik and Piskorski's analysis (37–42) and Porta's index (43, 69) were used. Guzik and Piskorski proposed two areas of HRA analysis: (i) study of the contributions (defined as the percentage of cumulative distance of points) of accelerations (_a_) and decelerations (_d_) to short-term (SD1) and long-term (SD2) variability and total variability (SDNN) (37–40) and (ii) analysis of monotonic runs of accelerations (AR), decelerations (DR) and neutral (NR) (39). For short-term variability, the authors defined C1_a_ and C1_d_ as relative contributions of accelerations (SD1_a_) and decelerations (SD1_d_) respectively to the short-term variance (SD1); for long-term variability: C2_a_ and C2_d_: relative contributions of accelerations (SD2_a_) and decelerations (SD2_d_) respectively to the long-term variance (SD2); and for total variability: C_a_ and C_d_: relative contributions of accelerations (SDNN_a_) and decelerations (SDNN_d_) respectively to total variance (42). HRA was present when the contributions of HR decelerations to short-term variability (Guzik's index—GI) were greater than those of accelerations (C1_d_ > C1_a_) and the contributions of accelerations to long-term variability were greater than those of decelerations (C2_a_ > C2_d_). Porta's index (PI) is based on the evaluation of the percentage of negative RRi (points below the line of identity) with respect to the number of overall points not on the line of identity. A PI < 50% means that decelerations are generally less numerous than accelerations (43, 69).

### 2.9 Ultra-short-term parameters

Special attention has been given by coaches and sports practitioners to (i) limit the time needed to obtain reliable physiological outcomes and (ii) look for parameters that can be used in the applied sports field (70, 71). In the present study, ultra-short-term (1 min): RespRate, HR, HR_min_, HR_max_, mRR, RMSSD, lnRMSSD. 0V and 2UV from all transformation methods, SD1 (SD1_d_, SD1_a_) and C1_d_, SD2 (SD2_d_, SD2_a_) and C2_d_, SDNN_d_, SDNN_a_ and C_a_, PI and deceleration and acceleration runs (1 to 5) were calculated for the fifth min of rest, the fifth min of SNSa stimulation, and the first, second and third mins of post-stimulation recovery.

### 2.10 Statistical analysis

The Shapiro–Wilk test was used to assess the normality of the data distribution. RRi stationarity was tested using the Augmented Dickey-Fuller test for each athlete's full RRi series. To compare HRV parameters obtained during different conditions, Friedman's repeated measures analysis of variance (ANOVA) by ranks followed by the Dunn–Bonferroni test for *post hoc* comparisons between pairs was used. To assess the correlation between changes in HRV parameter values between two measurements (SNSa stimulation—resting examination; post-stimulation recovery—SNSa stimulation) and changes in respiratory rate or changes in heart rate and professional career duration, Spearman's rank correlation coefficient was calculated. The threshold probability of *p* < 0.05 was used as the level of significance for all statistical tests. Statistical analyses were performed using PQStat Software (PQStat v.1.8.4.138, PQStat Software, Poznan, Poland). GraphPad Prism 5 (GraphPad Software Inc., San Diego, CA, USA, 2005) was used to generate figures.

## 3 Results

The results of 2 out of 11 athletes were excluded due to the diagnosis of non-sinus atrial rhythm (*n* = 1) and second-degree atrioventricular block (Wenckebach) (*n* = 1)—athletes were referred for medical evaluation. Consequently, results of 9 elite ski mountaineering athletes (6 female), medalists of the 206 World Cup, World Championships, European Championships, and National Championships were included in the statistical analysis. The mean (±SD) age, body mass, height, body mass index (BMI), and career duration were: 26 years (±7), 64 kg (±10), 172 cm (±10), 26 kg/m^2^ (±7) and 6.4 years (±4.2) respectively.

Lack of stationarity of the RRi data series was observed for 1 athlete (#8) at rest, 5 athletes (#2, #5, #6, #8, #9) during SNSa stim. and 4 athletes (#2, #3, #4, #9) during post-stimulation recovery. Individual characteristics, sports achievements, and RRi data series with stationarity assessment for each condition can be found in the Supplementary Materials (pages 1–6).

The results of the parameters calculated based on 5-min recordings from the analyzed conditions with *p*-values from Friedman's repeated measures analysis of variance by ranks and the results of the Dunn-Bonferroni post-hoc test are presented in Table 1. HR, HR_min,_ and RRi were significantly higher during SNSa stim. than during rest and significantly lower during post-stimulation recovery than during SNSa stim. Mean RRi was significantly lower during SNSa stim. than during rest and significantly higher during post-stimulation recovery than during SNSa stim. HR_max_, 0V (all methods except the Max-min method) and DR1 were significantly higher during SNSa stim. than during rest. There was a remarkable trend for other parameters. Numerically, values of RespRate, C1_a_, PI, DR3, DR4, DR5, AR1, AR3, AR4 and NR1 increased during SNSa stim. and decreased during post-stimulation recovery. Conversely, numerically, the values of RMSSD, lnRMSSD, pNN50, 2UV (all methods), SD1, SD1_d_, SD1_a_, C1_d_, and DR2 decreased during SNSa stim. and increased during post-stimulation recovery. The values of SDNN, SD2, SD2_d_, SD2_a_, SDNN_d_, SDNN_a_ and AR2 increased during SNSa stim. and then increased during post-stimulation recovery.

**Table 1:** Results of respiratory rate, heart rate, linear time-domain parameters, symbolic dynamics, and HRA analyses for all conditions.

Parameters		Rest	SNSa stim.	Post-stim. recovery	*p*-value
RespRate [breaths/min]		11 (7–17)	13 (10–23)	12 (6–17)	0.105
HR [bpm]		64 (53–71)	75 (67–78)**	64 (56–67)^††^	**<0** **.** **001**
HR_min_ [bpm]		59 (48–66)	63 (56–71)*	58 (48–62)^†††^	**<0** **.** **001**
HR_max_ [bpm]		71 (63–85)	86 (77–103)***	75 (71–98)	**<0** **.** **001**
Linear analysis					
RRi [number]		321 (269–354)	375 (337–391)**	322 (285–333)^††^	**0** **.** **001**
mRR [ms]		936 (849–1124)	801 (768–890)**	935 (901–1062)^††^	**0** **.** **001**
SDNN [ms]		62 (28–139)	68 (43–93)	73 (44–131)	**0** **.** **049**
RMSSD [ms]		54 (26–150)	36 (30–61)	66 (30–122)	0.062
lnRMSSD		4.0 (3.3–5.0)	3.6 (3.4–4.1)	4.2 (3.4–4.8)	0.062
pNN50 [%]		40 (6–73)	18 (9–55)	42 (9–67)	0.169
Symbolic dynamics					
σ	0V [%]	26 (6–37)	32 (13–47)**	30 (12–47)*	**<0** **.** **001**
	2UV [%]	11 (1–28)	10 (7–24)	12 (3–26)	0.459
Max-min	0V [%]	12 (4–28)	24 (5–42)	19 (8–42)	**0** **.** **045**
	2UV [%]	17 (7–26)	13 (6–23)	16 (6–24)	0.169
Eq. prob. (q = 4)	0V [%]	15 (8–34)	30 (9–45)*	22 (11–36)	**0** **.** **032**
	2UV [%]	13 (4–31)	9 (7–25)	16 (3–26)	0.097
Eq. prob. (q = 6)	0V [%]	7 (3–20)	18 (5–31)**	13 (5–21)*	**0** **.** **004**
	2UV [%]	22 (7–38)	15 (10–32)	20 (5–30)	0.368
HRA					
SD1 [ms]		38 (19–106)	26 (21–43)	47 (21–86)	0.062
SD2 [ms]		78 (35–174)	87 (56–127)	93 (58–177)	0.121
SD1_d_ [ms]		27 (14–89)	17 (15–31)	35 (15–69)	0.062
SD1_a_ [ms]		28 (12–57)	20 (15–30)	31 (15–52)^†^	**0** **.** **032**
SD2_d_ [ms]		56 (23–99)	60 (41–85)	66 (43–120)	**0** **.** **045**
SD2_a_ [ms]		55 (27–144)	63 (39–94)	66 (40–148)*	**0** **.** **016**
C1_d_		0.54 (0.42–0.71)	0.52 (0.40–0.57)	0.56 (0.46–0.80)^†^	**0** **.** **032**
C1_a_		0.46 (0.29–0.58)	0.48 (0.43–0.60)	0.44 (0.20–0.54)	0.097
C2_d_		0.48 (0.26–0.63)	0.48 (0.44–0.58)	0.46 (0.26–0.53)	0.895
C2_a_		0.52 (0.37–0.74)	0.52 (0.42–0.56)	0.54 (0.47–0.75)	0.895
SDNN_d_ [ms]		44 (19–87)	47 (31–63)	52 (32–90)	0.121
SDNN_a_ [ms]		43 (21–109)	49 (30–69)	52 (30–107)	**0** **.** **049**
C_d_		0.49 (0.39–0.57)	0.49 (0.45–0.56)	0.48 (0.33–0.53)	0.895
C_a_		0.51 (0.43–0.61)	0.52 (0.45–0.56)	0.52 (0.47–0.67)	0.895
PI [%]		48 (36–57)	49 (45–57)	48 (32–55)	0.169
DR1 [number]		13 (1–32)	28 (3–52)**	14 (1–41)	**0** **.** **006**
DR2 [number]		20 (7–64)	18 (9–52)	27 (7–55)	0.479
DR3 [number]		16 (3–33)	17 (9–43)	12 (3–38)^†^	**0** **.** **010**
DR4 [number]		3 (0–18)	8 (0–15)	3 (0–9)	0.157
DR5 [number]		0 (0–8)	1 (0–5)	0 (0–6)	1.000
AR1 [number]		11 (2–41)	24 (2–51)	12 (2–26)	**0** **.** **027**
AR2 [number]		24 (3–58)	26 (8–51)	35 (0–46)	0.895
AR3 [number]		12 (1–26)	14 (7–46)	13 (7–26)	0.971
AR4 [number]		5 (0–13)	8 (0–14)	2 (0–9)	0.058
AR5 [number]		3 (0–9)	2 (0–7)	1 (0–12)	0.911
NR1 [number]		2 (0–7)	5 (0–14)	2 (1–5)	0.158

RespRate, respiratory rate; HR, heart rate; mRR, mean RR intervals; SDNN, standard deviation of normal-to-normal RRi; RMSSD, root mean square of successive RR interval differences; ln, log transformation; pNN50, percent of RR intervals differing >50 ms from the preceding one; Eq. prob., equal-probability; PI, Porta's index, *p*-values from Friedman's repeated measures analysis of variance by ranks.

Bold values denote statistically significant results.

* *p* < 0.05.

** *p* < 0.01.

*** *p* < 0.001—compared to rest.

^†^ *p* < 0.05.

^††^ *p* < 0.01.

^†††^ *p* < 0.001—compared to SNSa stim. using the Dunn-Bonferroni test.

Compared to rest, statistically significant increases in HR, HR_min_, HR_max_, RRi, 0V (σ and Eq. prob. methods q = 4 and q = 6), and DR1 were observed during SNSa stim. Differences in 0V (σ and Eq. prob. methods q = 4 and q = 6) and DR1 between SNSa stim. and the rest were not correlated with the RespRate differences, HR differences, or professional career duration (Table 2). Differences in 0V (Max-min method), 2UV (Eq. prob methods—q = 4 and q = 6), SD1_d_, SD2_d_, SD2_a_ and AR1 between SNSa stim. and the rest were correlated with RespRate differences. Greater increases in RespRate during SNSa stim. were associated with greater increases in 0V, 2UV and AR1 and greater decreases in SD1_d_, SD2_d_, SD2_a_. Greater differences in 0V and AR1 were observed in athletes with a shorter professional career.

**Table 2:** Correlation analysis between SNSa stimulation—rest differences and post-stimulation recovery—SNSa stimulation differences in parameters, differences in respiratory rate and heart rate, and also professional career duration.

		SNSa stim.—Rest difference in RespRate	SNSa stim.—Rest difference in HR	Career duration
SNSa stim.—Rest difference in:	RMSSD	−0.60, *p* = 0.088	−0.52, *p* = 0.148	0.08, *p* = 0.830
	lnRMSSD	−0.60, *p* = 0.088	−0.46, *p* = 0.208	−0.02, *p* = 0.966
	σ 0V	−0.05, *p* = 0.898	0.21, *p* = 0.586	−0.46, *p* = 0.210
	σ 2UV	0.50, *p* = 0.170	0.46, *p* = 0.218	0.11, *p* = 0.780
	Max-min 0V	0.70, ***p* = 0.036**	0.66, *p* = 0.054	−0.76, ***p* = 0.016**
	Max-min 2UV	−0.12, *p* = 0.765	−0.04, *p* = 0.914	0.37, *p* = 0.327
	Eq. p. (q = 4) 0V	−0.22, *p* = 0.576	0.17, *p* = 0.664	−0.27, *p* = 0.484
	Eq. p. (q = 4) 2UV	0.70, ***p* = 0.036**	0.29, *p* = 0.454	−0.06, *p* = 0.881
	Eq. p. (q = 6) 0V	0.08, *p* = 0.831	0.31, *p* = 0.413	−0.47, *p* = 0.201
	Eq. p. (q = 6) 2UV	0.77, ***p* = 0.016**	0.18, *p* = 0.648	−0.18, *p* = 0.634
	SD1_d_	−0.73, ***p* = 0.025**	−0.52, *p* = 0.148	0.24, *p* = 0.527
	SD1_a_	−0.57, *p* = 0.112	−0.46, *p* = 0.208	−0.01, *p* = 0.983
	SD2_d_	−0.68, ***p* = 0.042**	−0.32, *p* = 0.400	−0.02, *p* = 0.966
	SD2_a_	−0.70, ***p* = 0.036**	−0.38, *p* = 0.313	0.12, *p* = 0.763
	C1_d_	−0.58, *p* = 0.099	−0.11, *p* = 0.779	0.37, *p* = 0.327
	C2_d_	0.35, *p* = 0.356	0.22, *p* = 0.571	−0.09, *p* = 0.813
	C_a_	−0.03, *p* = 0.932	−0.26, *p* = 0.496	0.02, *p* = 0.966
	DR1	0.31, *p* = 0.418	0.37, *p* = 0.329	−0.19, *p* = 0.632
	AR1	0.80, ***p* = 0.009**	0.63, *p* = 0.072	−0.71, ***p* = 0.031**
		Post-stim. recovery—SNSa stim. difference in RespRate	Post-stim. recovery—SNSa stim. difference in HR	Career duration
Post-stim. recovery—SNSa stim. difference in:	RMSSD	−0.90, ***p* = 0.001**	−0.49, *p* = 0.177	−0.03, *p* = 0.949
	lnRMSSD	−0.88, *p* = **0.002**	−0.64, *p* = 0.061	−0.16, *p* = 0.682
	σ 0V	−0.03, *p* = 0.932	−0.19, *p* = 0.620	0.34, *p* = 0.376
	σ 2UV	0.32, *p* = 0.406	0.22, *p* = 0.574	−0.16, *p* = 0.682
	Max-min 0V	0.32, *p* = 0.406	0.76, ***p* = 0.017**	0.74, ***p* = 0.023**
	Max-min 2UV	−0.03, *p* = 0.932	−0.34, *p* = 0.366	−0.29, *p* = 0.442
	Eq. p. (q = 4) 0V	−0.02, *p* = 0.966	0.32, *p* = 0.404	0.89, ***p* = 0.001**
	Eq. p. (q = 4) 2UV	0.67, ***p* = 0.049**	0.04, *p* = 0.915	−0.18, *p* = 0.650
	Eq. p. (q = 6) 0V	0.18, *p* = 0.637	0.52, *p* = 0.152	0.54, *p* = 0.135
	Eq. p. (q = 6) 2UV	0.43, *p* = 0.244	0.10, *p* = 0.797	0.03, *p* = 0.949
	SD1_d_	−0.90, *p* = **0.001**	−0.49, *p* = 0.177	−0.03, *p* = 0.949
	SD1_a_	−0.88, *p* = **0.002**	−0.72, ***p* = 0.029**	−0.26, *p* = 0.498
	SD2_d_	−0.63, *p* = 0.067	−0.86, ***p* = 0.003**	−0.18, *p* = 0.634
	SD2_a_	−0.85, *p* = **0.004**	−0.59, *p* = 0.097	0.04, *p* = 0.915
	C1_d_	−0.83, *p* = **0.005**	−0.18, *p* = 0.635	−0.24, *p* = 0.542
	C2_d_	0.52, *p* = 0.154	−0.15, *p* = 0.699	−0.20, *p* = 0.603
	C_a_	−0.17, *p* = 0.668	0.56, *p* = 0.116	0.50, *p* = 0.166
	DR1	0.17, *p* = 0.668	0.17, *p* = 0.667	0.18, *p* = 0.634
	AR1	0.63, *p* = 0.067	0.64, *p* = 0.061	0.82, ***p* = 0.007**

Bold values denote statistically significant results.

Compared to SNSa stim., significant decreases in HR, HR_min_, RRi, and DR3 and increases in mRR, SD1_a,_ and C1_d_ were observed during post-stimulation recovery. Differences in RMSSD, 2UV (Eq. prob. q = 4), SD1_d_, SD1_a_ SD2_a_ and C1_d_ between SNSa stim. and post-stimulation recovery were correlated with differences in RespRate (Table 2). A greater decrease in RespRate during post-stimulation recovery was associated with a greater increase in RMSSD, SD1_d_, SD1_a_ SD2_a,_ and C1_d_ and a greater decrease in 2UV (Eq. prob. q = 4). Greater decreases in 0V (Max-min method) and greater increases in SD1_a_ and SD2_d_ were associated with greater decreases in HR. Greater differences in 0V (Max-min and Eq. prob. q = 4 methods) and AR1 were observed in athletes with a longer professional career.

**Figure 1:**
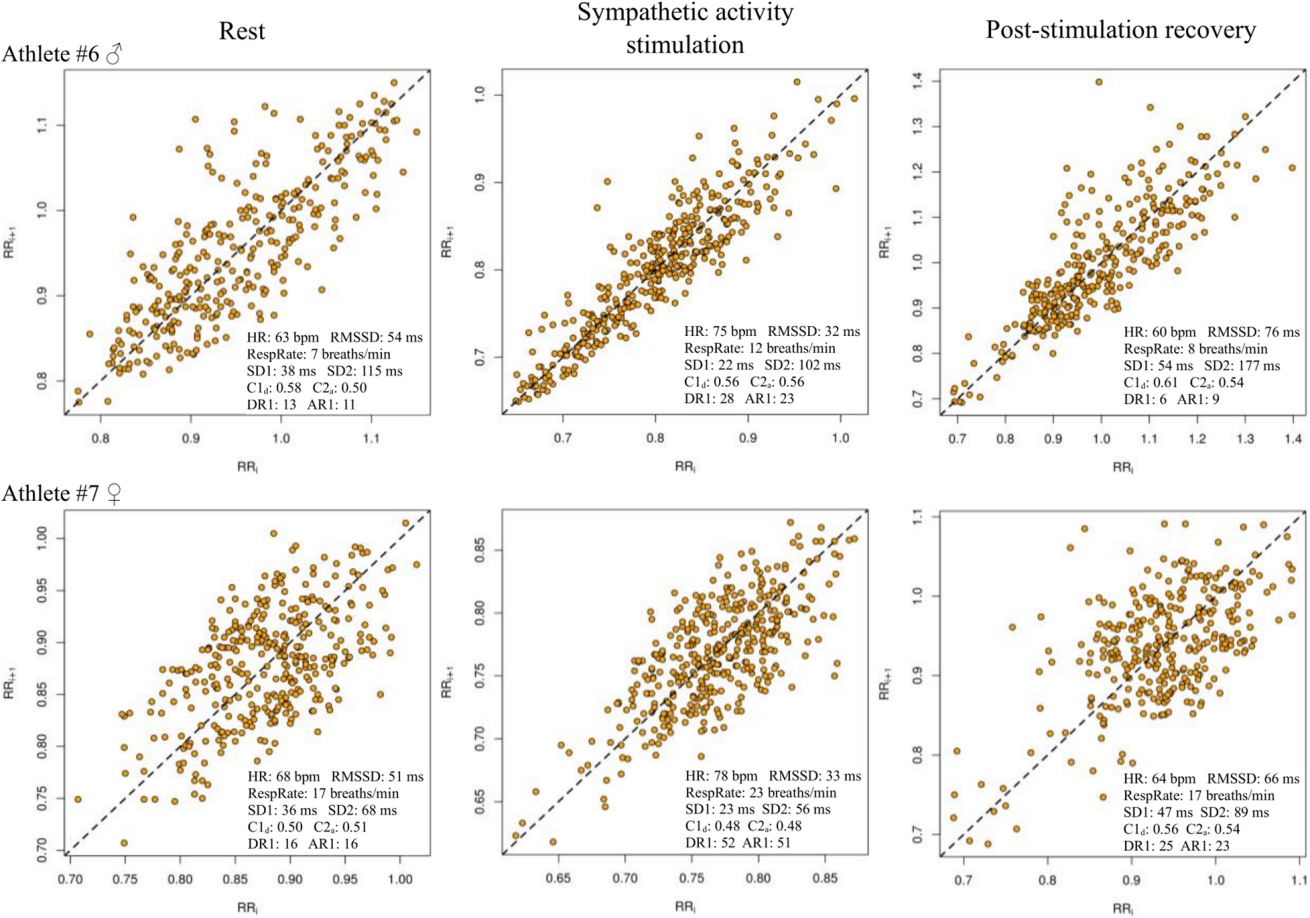
Poincaré plots for athletes #6 and #7 with results of HR, RespRate, RMSSD, SD1, SD2, C1_d_, C2_a_, DR1 and AR1 from the analyzed conditions.

Figure 1 shows Poincaré plots for athletes #6 and #7 (as examples) with the results of HR, RespRate, RMSSD, SD1, SD2, C1_d_, C2_d_, DR1 and AR1 from the analyzed conditions. It can be seen that the shape of the Poincaré plot is narrowed (confirmed in group analysis—Table 1, SD1) and shortened (in contrast to group analysis—Table 1, SD2) parallel to the line of identity during sympathetic activity stimulation.

The pattern of RRi changes with time from the fifth min of rest, the fifth min of SNSa stim., and the first, second, and third mins of post-stimulation recovery are shown in Supplementary Figure S1 (Supplementary Materials).

Supplementary Figure S2 (Supplementary Materials) shows the minute-by-minute changes in RespRate, HR, HR_min,_ and HR_max_ in all conditions. There was a different pattern of RespRate changes during the analyzed conditions, e.g., athlete #7 increased the rate of RespRate during the SNSa stim. compared to the rest condition whereas athlete #8 decreased the rate. HR increased progressively during SNSa stim. and decreased rapidly during the first min of post-stimulation recovery for all athletes.

The results of grouped analysis for ultra-short-term (1 min) parameters (Supplementary Table S1) from different conditions were similar to those calculated based on 5 min recordings. The values of ultra-short RespRate, HR, HR_min_, HR_max_, RRi, 0V (all methods), DR1, 4, 5, and AR1 were higher during the last min of SNSa stim. compared to the last min of rest and gradually decreased during the three minutes of post-stimulation recovery to values close to those of rest. The values of ultra-short RMSSD, lnRMSSD, 2UV (all methods), SD1, SD1_d_, SD1_a_, C1_d_, and SDNN_a_ decreased during the last min of SNSa stim. compared to the last min of rest and increased during the three mins of post-stimulation recovery to values even higher than those observed during the last min of rest. C_a_ values increased during the last min of SNSa stim. and then increased during post-stimulation recovery. Individual changes for ultra-short parameters calculated based on the fifth minute of rest, the fifth minute of SNSa stim., and the first, second, and third minutes of post-stimulation recovery with the repeated measures ANOVA and post-hoc analysis results are presented in Supplementary Figures S3–S6 (Supplementary Materials). Individual analysis provided additional information showing that some athletes may present different patterns of changes for HR dynamics and asymmetry, e.g., for athlete #4 during the last min of SNSa stim. the smallest nominal increases in RespRate and HR were observed with, surprisingly, increases in RMSSD, SD1_d,_ and C1_d_ and decreases in 0V; expected increases in HR, RMSSD, and 0V during the last min of SNSa stim. were observed with increases in SD2, SD2_d_ and SD2_a_ for athlete #1.

**Figure 2:**
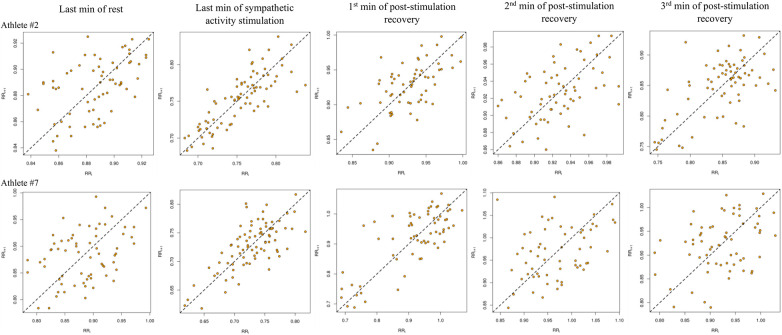
Poincaré plots generated based on ultra-short-term recordings of analyzed conditions for athletes #2 and #7.

Figure 2 shows Poincaré plots generated for athletes #2 and #7 (as examples) from the fifth minute of rest, the fifth minute of SNSa stim., and the first, second, and third minutes of post-stimulation recovery.

## 4 Discussion

Changes in measures of HR dynamics and asymmetry, calculated based on short-term non-stationary RRi time series induced by sympathetic stimulation and post-stimulation recovery, reflect sympathovagal shifts and are associated with exercise-induced alterations in respiratory rates and career duration in athletes who practice ski mountaineering.

The ability to modify cardiac autonomic activity may be an effective method for athletes in order to improve sports performance (6, 72) or short-term recovery (73, 74). Sympathetic predominance before and/or during competition has been shown to be helpful in extreme sports to achieve better results (6). Short-term (seconds, minutes) increased sympathetic tone and/or decreased parasympathetic outflow to the heart result in a progressive HR acceleration pattern (7, 75). However, HR cannot increase indefinitely, and the rate at which it increases or decreases is variable, i.e., periods of increasing or decreasing RRi are not equal (76). During sympathetic stimulation (or predominance), signal non-stationarity is interpreted as progressive RRi deceleration with a pattern characterized by more numerous tachycardic runs, tachycardic runs that are faster and longer (consisting of more beats) than bradycardic ones could be expected. In our study, short-term (5 min) and ultra-short-term (1 min) rates of patterns with no variations, number of acceleration runs of length 1, and short-term PI exhibited a tendency to be (significantly or nominally) greater whereas GI was smaller during sympathetic stimulation compared to rest examination. Athletes displayed a “comet” shaped Poincaré plot with visible asymmetry (the plot cloud above the identity line being larger than the plot cloud below the line) during the resting period. Asymmetry in Poincaré plots, which is commonly observed in most healthy individuals, suggests that HR accelerations operate in a different manner than decelerations, possibly due to baroreflex responses (36). A narrowed and shortened shape was obtained in athletes during sympathetic stimulation—this shape was similar to the “torpedo” shape observed in patients with heart failure (77). Our results are in line with previous findings in athletes (34) and healthy adults (43). In the mentioned studies, sympathetic stimulation was induced in modern pentathletes, identical to the present study, by short-term static isometric exercise (34), whereas in adults it was induced by a graded head-up tilt test (43). Our findings are similar to those obtained in response to acute mental stress in healthy students (78). Indeed, HRA was found to be sensitive to psychological influences—positive emotions produced a higher number of decelerations in short-term variability relative to total short-term variability than negative emotions (79). Recently, it has been suggested that Guzik's index may assess vagal withdrawal rather than sympathetic activation during the tilt maneuver (80).

The recovery period, which is crucial for preventing the accumulation of physical fatigue in athletes, could be optimized by controlling the individual's optimal breathing resonance and monitoring respiratory sinus arrhythmia (HRV biofeedback) (73). Regularization of breathing to a well-tolerated rate (i.e., 15 breaths/min) could stimulate efferent asymmetric autonomic patterns directed to the heart and/or might induce asymmetric responses of reflex cardiac control circuits such as the baroreflex (48). The GI increased and the number of acceleration runs of length 1 decreased during post-stimulation recovery to values exceeding those observed at rest. Recently, it has been shown that high-performance athletes manifest a greater number and magnitude of cardiac decelerations after an orthostatic challenge than non-athletes (30). In the present study, a greater increase in GI, but also vagally-mediated RMSSD, was associated with a greater decrease in breathing rate during post-stimulation recovery. On the other hand, a greater increase in respiratory rate from rest to short-term sympathetic stimulation was associated with greater increases in the rate of patterns with no variations from symbolic dynamics analysis (Max-min method) and the number of acceleration runs of length 1 but also with greater decreases in decelerations of short-term variance (SD1_d_), accelerations and decelerations of long-term variance (SD2_a_ and SD2_d_ respectively). Changes in SD1 and SD2 could be visually assessed by the shape of the Poincaré plot. The “comet” shaped plot with wider dispersion and visually more pronounced asymmetry during recovery than during the rest period was observed, e.g., in athlete #6 (Figure 1).

Klintworth et al. showed that Guzik's and Porta's indices were higher during a symmetrical breathing pattern (inspiration and expiration controlled in a 1:1 ratio) compared to a physiological pattern (1:2 ratio) in young healthy volunteers in the supine position at 0.22 Hz breathing (46). This was confirmed for 0.25 Hz breathing in the sitting position by Wang et al. who also showed that both indices were greater during 0.10 Hz breathing than during 0.25 Hz breathing in the supine and sitting positions (47). The duration of cardiac cycles during a single breath varies, i.e., RRi are longer during expiration (grouped bradycardic runs) and shorter during inspiration (grouped tachycardic runs) (48, 81, 82) due to the inhibited activity of cardiac vagal efferent fibers during inspiration (47). Moreover, the duration of inspiration and expiration during spontaneous breathing is asymmetrical—in healthy humans, the expiratory phase lasts longer (39, 40). However, as the breathing rate increases, both the expiratory and inspiratory times are shortened (47)—the maximum RRi during expiration is decreased because there is less acetylcholine released, while the minimum RRi during inspiration is increased because the released acetylcholine is not completely hydrolyzed (83). At higher breathing rates and lower tidal volumes there is less stimulation of the sympathoinhibitory reflex elicited by lung stretch (84, 85). An analysis of the association between changes in HR microstructure and breathing depth (or tidal volume) under different conditions would be an interesting idea for future research in athletes. However, the method used to measure tidal volume (e.g., by using a mouthpiece) could affect HRV data (86). Therefore, we are trying to implement Pneumonitors—portable devices that record breathing patterns using impedance pneumography along with single-lead ECG, subject motion, and/or pulse oximetry—into our practice (87, 88). Recently, they have been validated in children with heart disease in the supine position (89). Pneumonitors can also be utilized to estimate cardiorespiratory coupling strength, the “combined” measures characterizing cardiac regulation genuinely driven by respiration (90).

More attention to individual analysis seems to be necessary when assessing physiological responses to specific interventions in athletes (91, 92). Group analysis revealed an increase in respiratory rate, HR, rate of patterns with no variations, and number of grouped acceleration runs during sympathetic stimulation. Nevertheless, individual analysis showed that marginal increases in ultra-short-term (1 min) respiratory rate and HR during sympathetic stimulation were associated with expected marginal alterations in RMSSD, SD1, SD2, DR1, and AR1 but also with an unexpected decrease in the rate of patterns with no variations (Athlete #4 in Supplementary Figures S2–S5). Despite the changes in HR microstructure observed during short-term sympathetic stimulation and post-stimulation recovery, HRA, based on group analysis, was present in all conditions. On an individual basis, HRA was observed in 67%, 56%, and 78% of athletes during short-term rest, sympathetic stimulation, and post-stimulation recovery, respectively. In our opinion, analysis of changes in HR microstructure parameters and visual assessment of the Poincaré plot in individual athletes may complement the evaluation of cardiac autonomic nervous system modulation during specific sports tasks especially when changes in standard time-domain HRV parameters are lower than the smallest meaningful change.

The physiological response to an external stimulus depends on the type of stimulus and may depend on the athlete's sports experience (93, 94). Isometric handgrip exercises remain a well-studied test of sympathetic function (54, 95). The HR response to sustained handgrip exercises was sensitive to changes in workload throughout the season and has therefore been suggested to be useful in preventing excessive accumulation of training-induced fatigue in collegiate female swimmers (96). Abaji et al. observed significantly lower vagally-mediated high-frequency power calculated based on the 3 min recordings during isometric handgrip contraction (30% MVC) in post-concussion athletes than in the control group (97). In future studies, our data may aid in the identification of abnormal responses (as well as recovery) in fatigued or concussed athletes. Continuously squeezing the dynamometer's handle at 30% of maximal voluntary contraction proved to be an optimal dose to observe a gradual increase in HR during the first min and five mins of stimulation in most athletes. Importantly, different HR dynamics and asymmetry patterns in response to sympathetic stimulation and during post-stimulation recovery were observed in those with different professional career durations. Greater increases in the acceleration runs of length 1 and the rate of patterns with no variations (Max-min method) during sympathetic stimulation were associated with a shorter career duration. Greater decreases in these parameters during post-stimulation recovery were associated with longer careers.

The small sample size, cross-sectional study design, and data obtained during supine ECG recordings performed in controlled laboratory settings should be recognized as limitations of this pilot study. Limited symbolization strategy was used. Nevertheless, Porta et al. suggested that adopting different symbolization strategies may not have had an important impact on the conclusions (98).

## 5 Conclusions, clinical implications, and future directions

Analysis of changes in HR dynamics and asymmetry indices could be useful to detect sympathovagal shifts during short-term sympathetic activity stimulation and post-stimulation recovery in athletes who practice ski mountaineering. Visual detection of narrowed and shortened parallels to the line of identity shape of the Poincaré plot could indicate sympathetic predominance. Importantly, the physiological response was more pronounced in less experienced athletes.

A mobile application that continuously displays real-time outcomes of RMSSD [recommended in sport science (9)], percentage ratio of rate of a specific pattern with different variations, Poincaré plot with variance-based HR asymmetry descriptors with number of monotonic runs of accelerations and decelerations, and respiratory rate would have the potential to improve the recognition of individual athletes' cardiac autonomic activity during different activities in the field.

The present study is a pilot exploratory research project by our team. In the future, we will study the agreement between ultra-short-term and short-term HR dynamics and asymmetry, test-retest repeatability of HRA, changes in HR complexity during aerobic and anaerobic fitness tests, along with information on the mechanics of breathing in different groups of amateur and professional athletes.

## Data Availability

The raw data supporting the conclusions of this article will be made available by the authors, without undue reservation.
