# Supplementary material for: Heart rate dynamics and asymmetry during sympathetic activity stimulation and post-stimulation recovery in ski mountaineers—a pilot exploratory study
Source: Front Sports Act Living. 2024 Feb 29;6:1336034. doi: 10.3389/fspor.2024.1336034 (PMC10941344; doi:10.3389/fspor.2024.1336034)
Supplement: Supplementary file 1 [file Table1.docx]

Supplementary Materials

Jakub S. Gąsior*, Maciej Gąsienica-Józkowy, Marcel Młyńczak, Maciej Rosoł, Robert Makuch, Rafał Baranowski, Bożena Werner

*** Correspondence:** Jakub S. Gąsior [jakub.gasior@wum.edu.pl](mailto:jakub.gasior@wum.edu.pl)

**Individual characteristics, sports achievements, RRi data series with stationarity assessment, one-minute RRi data series**

Athlete #1 ♀, 25 years, 47 kg, 153 cm, participation in: World Cup, World and European Championships

Athlete #2 ♂, 16 years, 71 kg, 185 cm, participation in: National Championships, National Cup

Athlete #3 ♂, 19 years, 69 kg, 180 cm, participation in: World Cup, European and National Championships, National Cup

Athlete #4 ♀, 30 years, 65 kg, 168 cm, participation in: National Championships, National Cup

Athlete #5 ♀, 40 years, 54 kg, 167 cm, participation in: National Championships, National Cup

Athlete #6 ♂, 27 years, 74 kg, 182 cm, participation in: World and National Championships, National Cup

Athlete #7 ♀, 16 years, 50 kg, 162 cm, participation in: National Championships, National Cup

Athlete #8 ♀, 30 years, 70 kg, 178 cm, participation in: World Cup, World, European and National Championships, National Cup

Athlete #9 ♀, 28 years, 73 kg, 172 cm, participation in: World Cup, European and National Championships


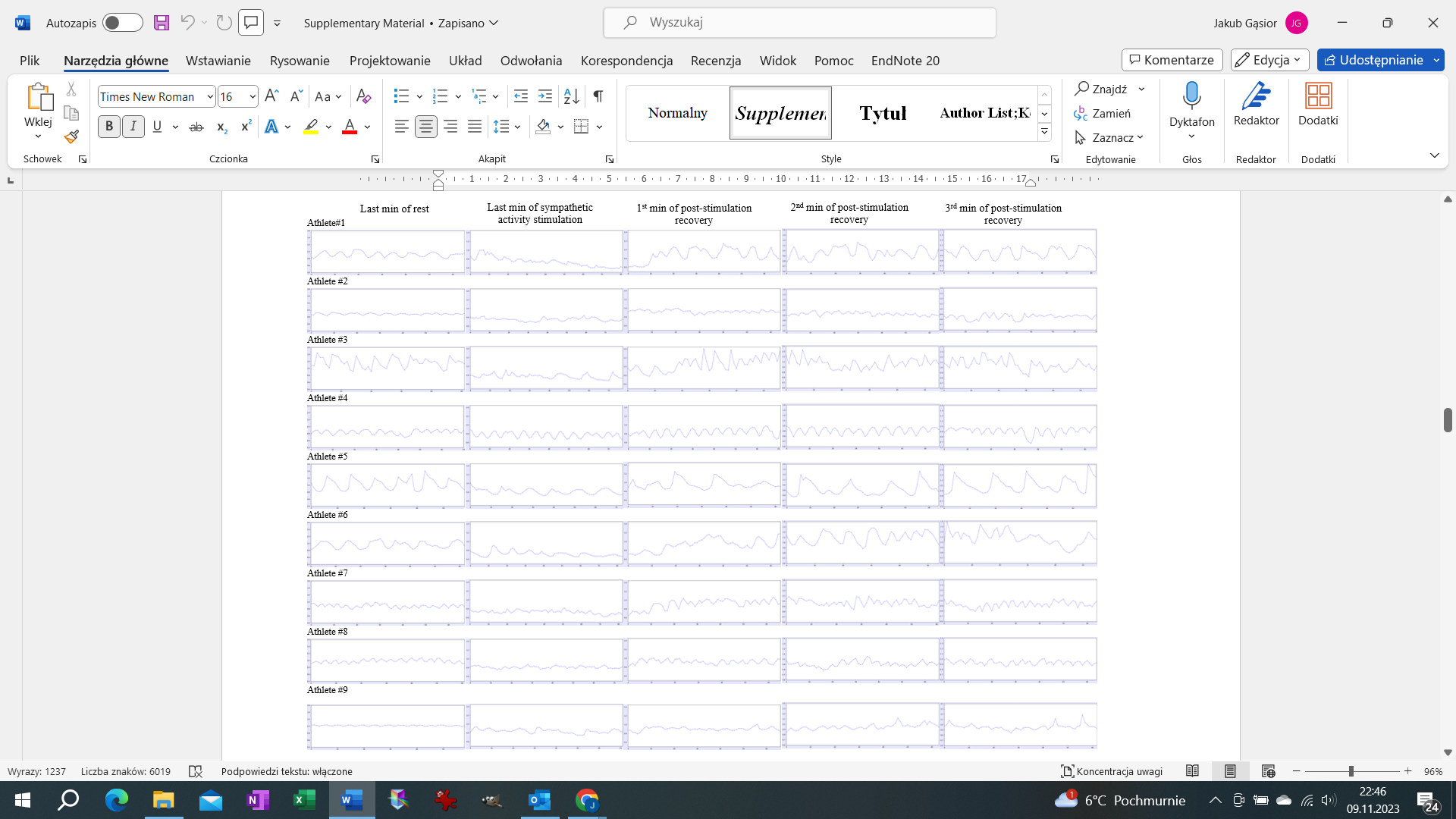


**Figure S1**. Pattern of RRi changes from the 5^th^ min of rest, the 5^th^ min of SNSa stim., 1^st^, 2^nd^ and 3^rd^ min of recovery; x-axis – number of RRi, y-axis – RRi in seconds


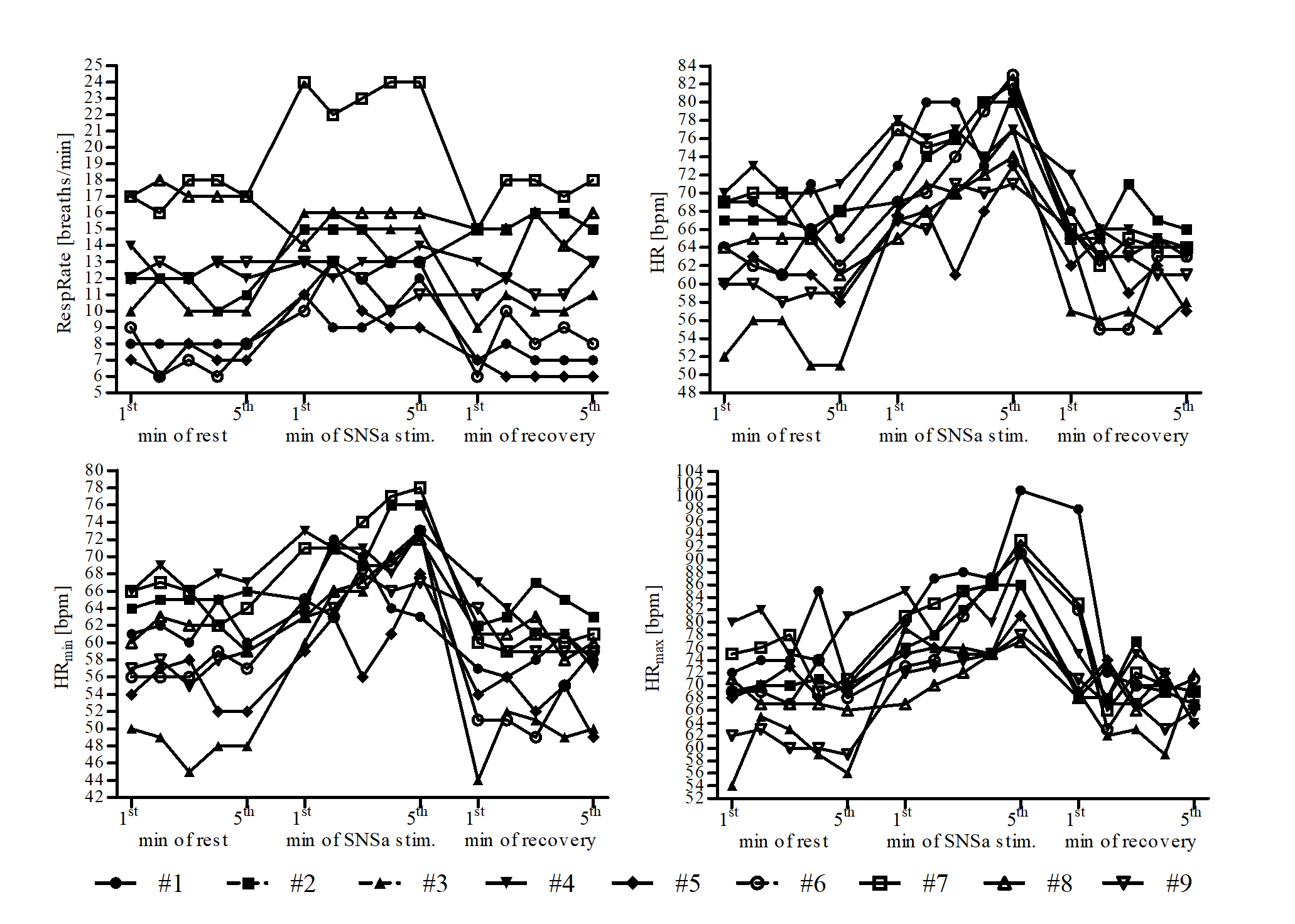


**Figure S2**. Minute-by-minute changes in RespRate, HR, HR_min_ and HR_max_ in all conditions for all considered participants.

**Table S1**. Results of respiratory rate, heart rate, selected linear time-domain parameters, symbolic dynamics and HRA analyses for the fifth minute of rest, fifth minute of SNSa stimulation, first, second and third minute of post-stimulation recovery.

| **Parameters** | | **Last min of rest** | **Last min of SNSa stim.** | **1^st^ min of post-stim. recovery** | **2^nd^ min of post-stim. recovery** | **3^rd^ min of post-stim. recovery** |
| --- | --- | --- | --- | --- | --- | --- |
| RespRate [breaths/min] | | 11 (7–17) | 13 (9–24) | 11 (6–15) | 11 (6–18) | 11 (6–18) |
| HR [bpm] | | 62 (51–71) | 77 (71–83) | 65 (57–72) | 63 (55–66) | 63 (55–71) |
| HR_min_ [bpm] | | 59 (48–67) | 73 (63–78) | 60 (44–68) | 59 (51–63) | 59 (50–68) |
| HR_max_ [bpm] | | 69 (56–81) | 86 (77–102) | 71 (68–98) | 68 (60–74) | 68 (64–78) |
| RRi [number] | | 63 (52–71) | 78 (72–84) | 66 (57–72) | 64 (55–67) | 63 (56–71) |
| mRR [ms] | | 965 (847–1169) | 776 (723–845) | 923 (836–1060) | 950 (906–1097) | 957 (848–1083) |
| RMSSD [ms] | | 55 (18–176) | 31 (23–53) | 67 (28–159) | 66 (32–108) | 65 (41–117) |
| lnRMSSD | | 4.0 (2.9–5.2) | 3.4 (3.1–4.0) | 4.2 (3.3–5.1) | 4.2 (3.5–4.7) | 4.2 (3.7–4.8) |
| σ | 0V | 20 (2–39) | 28 (5–56) | 30 (5–48) | 17 (3–49) | 25 (3–47) |
|  | 2UV | 9 (2–30) | 8 (1–21) | 14 (3–20) | 13 (2–32) | 14 (0–27) |
| Max-min | 0V | 4 (0–16) | 19 (0–50) | 13 (4–34) | 9 (0–27) | 7 (2–34) |
|  | 2UV | 25 (7–42) | 13 (0–26) | 22 (8–27) | 19 (6–35) | 16 (7–35) |
| Eq. p.  (q=4) | 0V | 13 (0–21) | 28 (4–51) | 23 (5–60) | 14 (2–43) | 15 (3–31) |
|  | 2UV | 14 (5–29) | 8 (2–21) | 13 (3–31) | 13 (0–30) | 17 (2–37) |
| Eq. p.  (q=6) | 0V | 2 (0–20) | 17 (0–36) | 13 (2–27) | 6 (0–14) | 8 (2–13) |
|  | 2UV | 20 (5–38) | 17 (8–27) | 22 (6–32) | 24 (5–35) | 25 (7–33) |
| SD1 [ms] | | 38 (13–126) | 22 (16–37) | 47 (20–113) | 47 (23–75) | 46 (29–83) |
| SD2 [ms] | | 79 (16–196) | 66 (33–145) | 129 (43–198) | 68 (39–157) | 84 (44–176) |
| SD1_d_ [ms] | | 27 (9–106) | 17 (11–26) | 37 (13–93) | 36 (16–58) | 33 (20–77) |
| SD1_a_ [ms] | | 27 (10–68) | 15 (12–27) | 25 (15–65) | 30 (16–47) | 32 (20–45) |
| SD2_d_ [ms] | | 58 (13–111) | 45 (25–106) | 64 (28–144) | 52 (29–94) | 49 (32–118) |
| SD2_a_ [ms] | | 53 (9–162) | 49 (22–99) | 97 (32–163) | 58 (26–125) | 68 (30–149) |
| C1_d_ | | 0.54 (0.43–0.71) | 0.52 (0.42–0.64) | 0.57 (0.44–0.73) | 0.59 (0.43–0.75) | 0.54 (0.43–0.85) |
| C2_d_ | | 0.54 (0.26–0.63) | 0.51 (0.42–0.57) | 0.49 (0.25–0.55) | 0.49 (0.27–0.58) | 0.41 (0.28–0.58) |
| SDNN_d_ [ms] | | 45 (11–98) | 34 (19–76) | 53 (23–106) | 41 (24–74) | 42 (27–88) |
| SDNN_a_ [ms] | | 43 (9–122) | 37 (18–71) | 72 (25–124) | 46 (22–91) | 53 (28–108) |
| C_a_ | | 0.47 (0.42–0.61) | 0.49 (0.45–0.57) | 0.51 (0.45–0.70) | 0.51 (0.45–0.63) | 0.55 (0.44–0.62) |
| PI [%] | | 49 (32–58) | 47 (38–55) | 49 (34–52) | 48 (38–58) | 46 (31–56) |
| DR1 | | 2 (0–10) | 5 (0–13) | 5 (0–9) | 3 (0–9) | 3 (1–11) |
| DR2 | | 4 (0–14) | 4 (1–10) | 4 (1–9) | 5 (1–8) | 4 (1–16) |
| DR3 | | 3 (0–7) | 2 (1–10) | 2 (1–10) | 3 (0–11) | 3 (0–5) |
| DR4 | | 0 (0–4) | 2 (0–3) | 1 (0–4) | 1 (0–3) | 0 (0–2) |
| DR5 | | 0 (0–2) | 0 (0–2) | 0 (0–2) | 0 (0–1) | 0 (0–1) |
| AR1 | | 2 (0–5) | 6 (0–14) | 3 (1–5) | 2 (0–5) | 2 (0–8) |
| AR2 | | 5 (0–11) | 4 (0–8) | 4 (0–10) | 7 (1–12) | 6 (0–11) |
| AR3 | | 3 (0–5) | 3 (0–9) | 4 (0–8) | 3 (1–4) | 3 (0–5) |
| AR4 | | 0 (0–3) | 1 (0–2) | 1 (0–2) | 0 (0–2) | 1 (0–4) |
| AR5 | | 0 (0–3) | 1 (0–3) | 1 (0–2) | 0 (0–2) | 0 (0–1) |

RespRate – respiratory rate, HR – heart rate, mRR – mean RR intervals, SDNN – standard deviation of normal-to-normal RRi, RMSSD – root mean square of successive RR interval differences, ln – log transformation, pNN50 – percent of RR intervals differing >50 ms from the preceding one, Eq. prob. – Equal-probability, PI – Porta’s Index


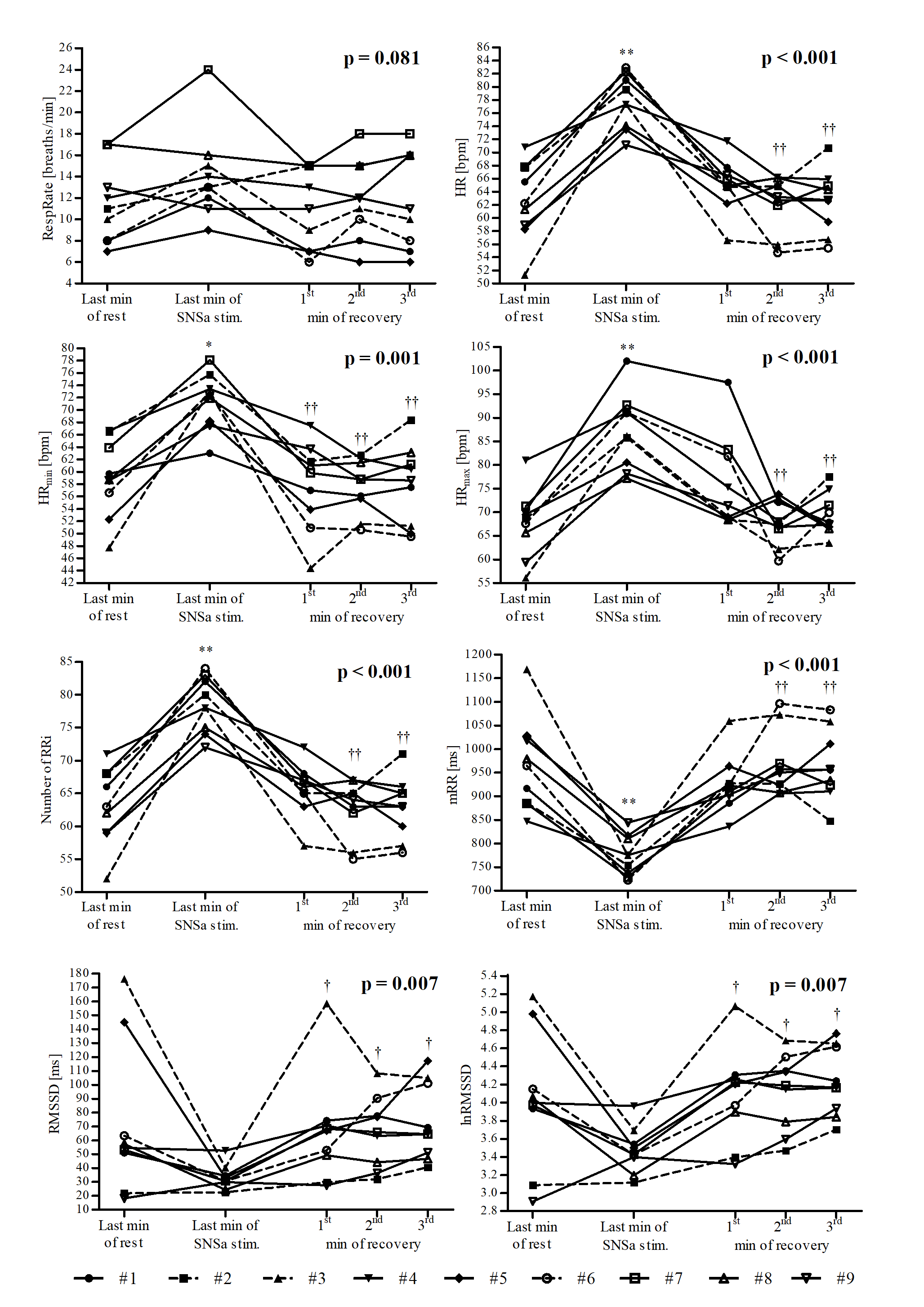


**Figure S3**. Individual changes for ultra-short respiratory rate, HR and time domain HRV parameters calculated based on the fifth min of rest, the fifth min of SNSa stim., first, second and third min of post-stimulation recovery with ANOVA and post-hoc analysis results. * p < 0.05, ** p < 0.01 – compared to last minute of rest using Dunn-Bonferroni test, † p < 0.05, †† p < 0.01 – compared to last minute of SNSa stim. using Dunn-Bonferroni test


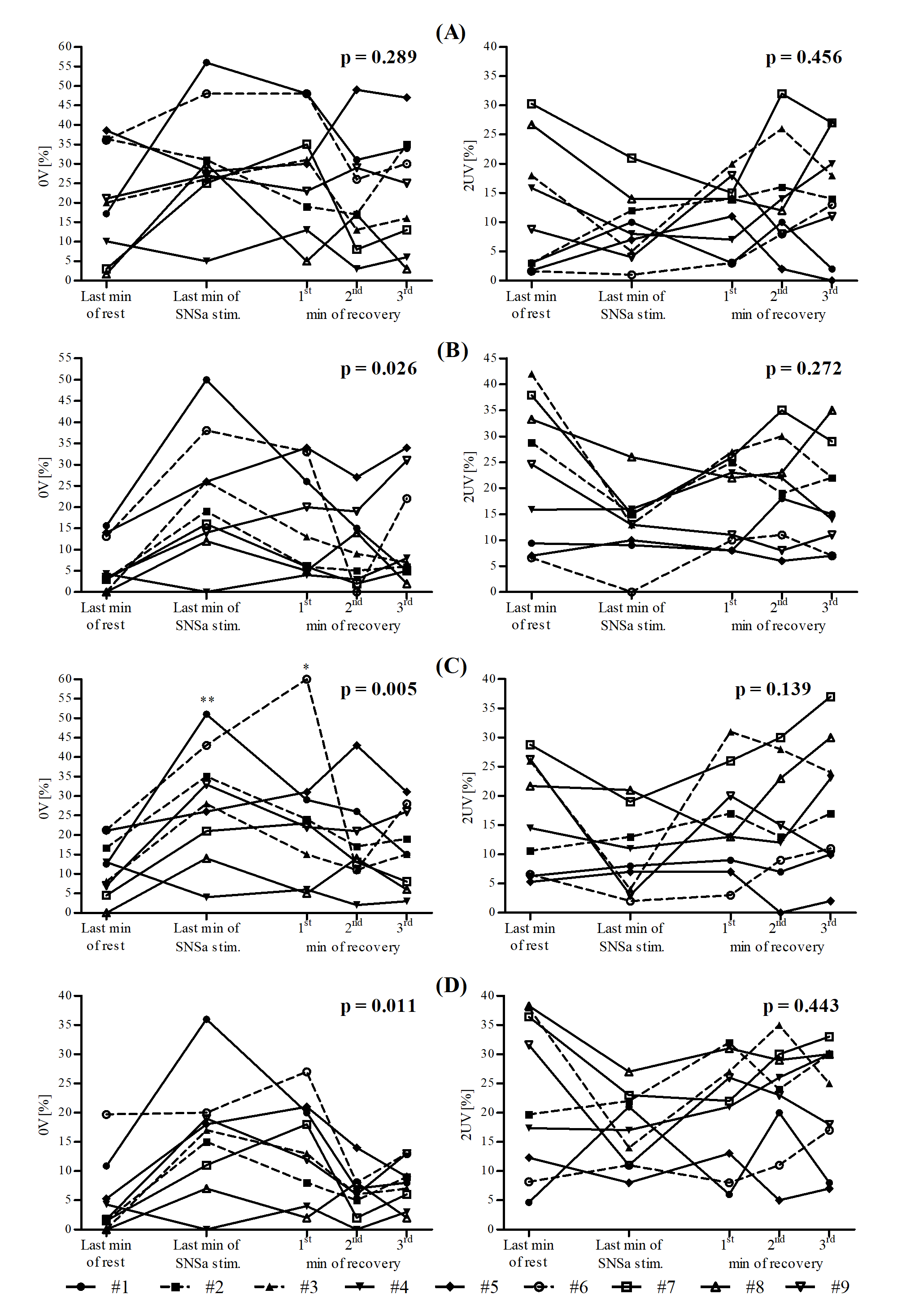


**Figure S4**. Individual changes for ultra-short symbolic dynamics indices calculated based on the fifth min of rest, the fifth min of SNSa stim., first, second and third min of post-stimulation recovery with ANOVA and post-hoc analysis results, * p < 0.05, ** p < 0.01 – compared to last minute of rest using Dunn-Bonferroni test (A) σ method, (B) Max–min method, (C) Equal-probability method (q=4), (D) Equal-probability method (q=6)


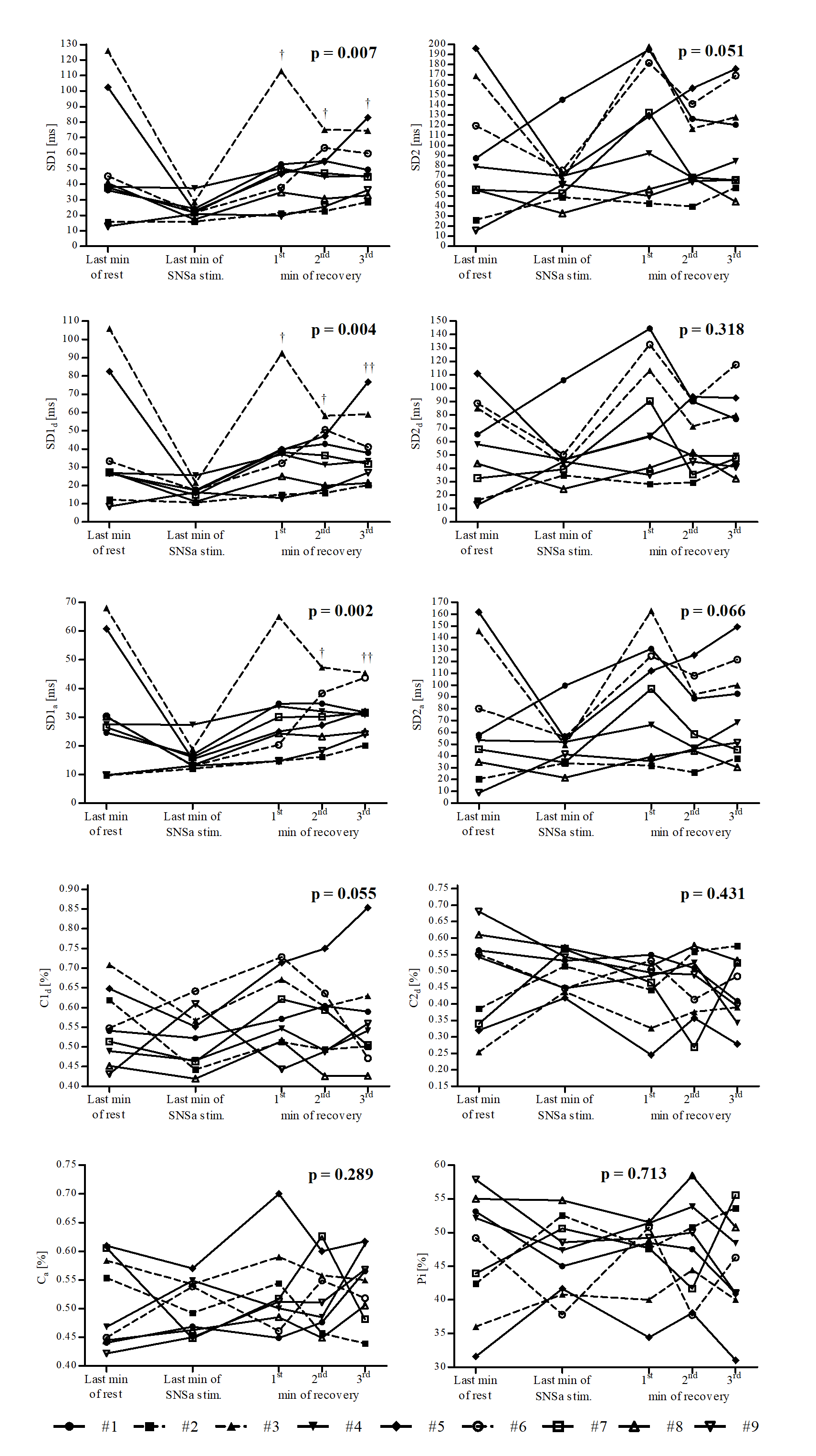


**Figure S5**. Individual changes for ultra-short HRA calculated based on the fifth min of rest, the fifth min of SNSa stim., first, second and third min of post-stimulation recovery with ANOVA and post-hoc analysis results, † p < 0.05, †† p < 0.01 – compared to last minute of SNSa stimulation using Dunn-Bonferroni test


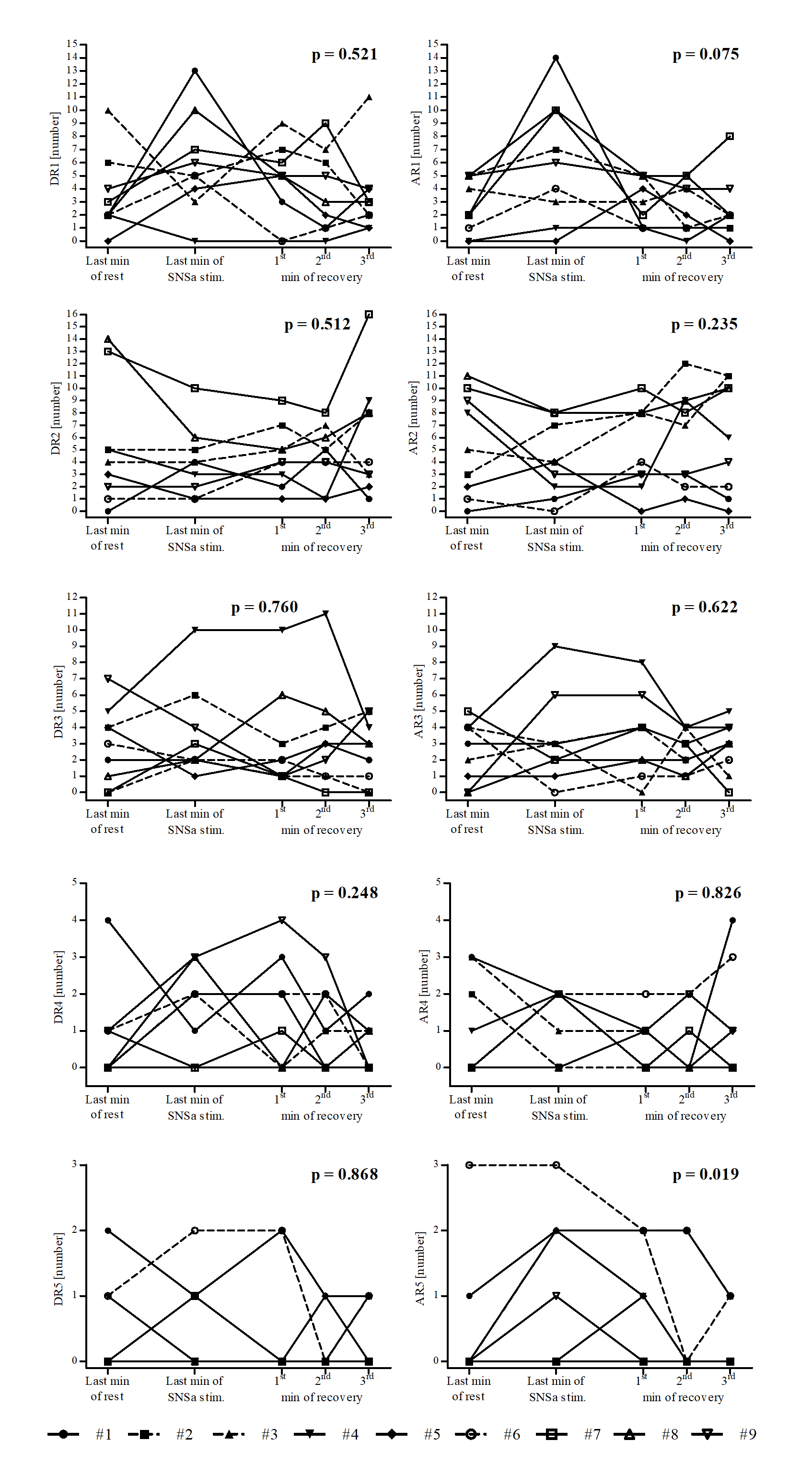


**Figure S6**. Individual changes for ultra-short monotonic runs calculated based on the fifth min of rest, the fifth min of SNSa stim., first, second and third min of post-stimulation recovery with ANOVA and post-hoc analysis results
